# Microbes That Endanger Health

**Published:** 1887-12

**Authors:** 


### MICROBES THAT ENDANGER HEALTH.

There is a world of living organisms, both vegetable and animal, which only the microscope can reveal to us. But, while the unaided eye cannot detect them, they are for that very reason the more dangerous to health and life. The spores or seeds from which microbes originate, are like the seeds and eggs of vegetable and animal life of a larger growth in most of the laws which govern their development and growth. Like weed seeds and insect eggs, which are found wherever there are favorable conditions for their germination, so these microbic spores are found in every conceivable place where there is likely to be a chance for germination. It is also true that the different species are just as distinct, and reproduce after their own image as faithfully as animals and vegetables of a larger growth. Again, the incubation of these spores requires the same conditions of moisture and heat for their germination. Place a grain of wheat in any place where it is perfectly dry, and it will never be quickened into life, but as soon as it comes in contact with the combined influences of moisture and heat, it will germinate. It is still a matter of some scientific doubt whether any vegetable or animal matter will putrefy unless it contains living spores.

It seems to be a proven fact that water can be so treated by the fumes of burning sulphur, as to kill every spore, and then in the water, thus purified fruit can be kept for a great length of time. This method was exhibited and explained at a late fair in the Mechanics Building, Boston. This, if it is a fully proven fact, goes far toward supporting the theory that all putrefactive processes are occasioned by the germination of spores. However this may be, the fact remains that anything which can putrefy, contains and will develop microbes.

It is rightly estimated that about one-half the diseases, especially of civilization, arise from unsanitary surroundings, or, in plain terms, from filth!

There are places in Africa where vegetation is so rank, and the heat so intense and prolonged that it is fatal to sleep there, even for one night. Thousands of men were sacrificed to the moloch of malaria, in building the railroad across the Isthmus of Panama. The poor victims were as numerous as the ties on the railroad track.

The building of the De Lesseps canal is only a little less fatal, from the fact that more natives are employed than formerly. Millions of people, some near to us by ties of blood and affection, have been martyrs to western malaria as they have turned up the virgin soil of the prairies, full of vegetable matter, which by the resulting putrefactive process, breeds fevers and death. After the vegetable mold has thoroughly decayed, there is a marked abatement of malarial diseases. People who live in the extreme South, near great undrainable swamps and stagnant bayous, will always be in danger of fevers. Frost will kill most forms of microbic life if brought directly in contact with the freezing temperature Cholera, small-pox and certain other zymotic germs seem to outlive the winter, probably because they are intrenched in warm houses. It is a cause for devout gratitude that scientists and physiologists are giving special and increased attention to this subject. When we drain our swamps or shun them as places of residence, and when we have perfect sewerage systems, then the golden period of freedom from zymotic diseases will be near.

An old plumber affirms that one-half the plumbing in cities is defective to-day. Many of the pipes are old, and the soil, in places, has settled in a way to break them. It is said that the city of Canton, China, with nearly two millions of inhabitants, packed together over about the same area as Boston, is almost free from contagious diseases, from the fact that every day the garbage and excrements are carried away to the farms for fertilizing purposes. If we must have a water system of sewerage we certainly should have better plumbing and trapping.

The cholera microbe, whose native habitat is in Asia, is a fellow of fearful vitality. As we trace the recent outbreak of cholera back to its evident origin, we are compelled to exclaim, "Behold how great a matter a little fire kindleth!" There is no reasonable doubt but what all the mischief had its origin in a miserable little village in Egypt, near one of the mouths of the River Nile, called "Damietta." An epidemic caused a large number of deaths among their cattle. Instead of burying them in the soil, nature's own regenerating alchemy, they dumped them into fresh water, where, under the festering influence of a tropical sun, they soon started a cholera plantation. From this village the fatal disease spread over Egypt, then into Asia and Europe, and for years past has kept the whole civilized world in dread and anxiety.

What we need, and we trust we shall soon have, is an international health commission that will watch these plague spots of the earth, and prevent the beginning of what is so widespread and fatal in its results.

But until sanitary science and practice have secured safety we can only avoid, so far as possible, unwholesome surroundings and also have at hand some malarial antidote to apply at the beginning of trouble in that regard. Persons in robust health are comparatively safe from malarial attack, unless living where the most unhealthy conditions exist. It is doubtful whether microbes can reproduce themselves in blood which is in its normal and healthy temperature. But let the temperature be raised by a cold, or by extreme exhaustion, and then the blood becomes a hot-bed for bacterial propagation. Again, children are much more liable to feel the effects of unsanitary conditions than adults. Nearly one-half of our people die before they are seventeen years of age, and the diseases of childhood are almost entirely of the zymotic, or contagious, filth type, a mild malaria, which to an adult, may be comparatively harmless, may prove fatal to a child.

But we have, as yet, only touched the outer circle of prevention.

Certainly it is high time to attend to a matter which is as vital to the weal of our city as the rum-drinking problem. But the light is increasing and better days are at hand. T. P. B.

